# Medication-Induced Factor V Inhibition in the Setting of Refractory Coagulopathy

**DOI:** 10.3390/hematolrep14040041

**Published:** 2022-09-21

**Authors:** Brandon Travis Wiggins, Daniel Ramirez, Daniel Taylor, William Reichardt, Alyssa Kipke, Mark Minaudo

**Affiliations:** 1 Department of Internal Medicine, Ascension Genesys Hospital, Grand Blanc, MI 48439, USA; 2 Division of Gastroenterology, Department of Internal Medicine, Ascension Genesys Hospital, Grand Blanc, MI 48439, USA; 3 Division of Hematology and Oncology, Department of Internal Medicine, Ascension Genesys Hospital, Grand Blanc, MI 48439, USA

**Keywords:** coagulopathy, acquired factor V inhibitor, cirrhosis

## Abstract

Liver cirrhosis is commonly associated with coagulopathies, typically demonstrated by elevated prothrombin time, international normalized ratio, and partial thromboplastin time. In the setting of bleeding related to coagulopathies, oftentimes physicians try to reverse coagulopathy through a variety of methods including the use of vitamin K and fresh frozen plasma. Rarely, attempts at reversing coagulopathy are unsuccessful due to severe disease or factor inhibitors. The treatment of acquired factor V inhibitors is primarily performed through immunosuppression and supportive care for the initial bleeding episode. Early detection and treatment of factor V inhibition is challenging in a setting of underlying cirrhosis-related coagulopathy.

## 1. Introduction

Acquired Factor V inhibition is a rare, but potentially life-threatening autoimmune condition where antibodies acting against the clotting protein factor V can cause a bleeding diathesis that is refractory to supportive care [1]. Hemorrhagic symptoms for acquired factor inhibition are like those of cirrhosis-related coagulopathy, so missing or delaying the diagnosis is a life-threatening concern [2].

Most cases of acquired factor V inhibition are described after exposure to fibrin glues or bovine thrombin preparations that contain bovine factor V [3]. For patients that do not have such exposures, there have been case reports of acquired factor V inhibition in patients with autoimmune disease, underlying malignancy, or postpartum state [3]. In some cases, the etiology is found to be drug-induced, with medications such as Rifaximin, direct thrombin inhibitors, and beta-lactam antibiotics [4,5].

Herein we describe a case of refractory coagulopathy in the setting of cirrhosis due to acquired factor V inhibition.

## 2. Case Presentation

An 83-year-old male with the past medical history of non-alcoholic steatohepatitis, alcoholic liver cirrhosis, chronic kidney disease, type 2 diabetes, coronary artery disease and heart failure presented to the emergency department after a fall at home. The patient was also complaining of mild abdominal pain with distention and melena for one week.

Laboratory work-up and vitals in the emergency department were remarkable for hemoglobin of 4.2 g/dL, international normalized ratio (INR) of 5, and platelet count of 89 K/cm^2^. Fibrinogen levels were ordered and within normal limits at 336 mg/dL. Computed tomography (CT) of the abdomen and pelvis demonstrated a large amount of ascites and liver cirrhosis. The patient was started on high dose IV proton pump inhibitors, octreotide, ceftriaxone, 15 mg of IV Vitamin K and received 2 units of packed red blood cells with 2 units of fresh frozen plasma (FFP). The patient was restarted on home lactulose and rifaximin to prevent hepatic encephalopathy. Gastroenterology was consulted and the patient was admitted to the floor. Hematology was also consulted considering the patient’s pancytopenia and coagulopathy.

The patient underwent an upper endoscopy and was found to have portal hypertensive gastropathy, hiatal hernia, gastric antral deformity with adherent clot and active oozing throughout the antrum of the stomach as well as the first and second portions of the duodenum. The patient received argon plasma coagulation, but there was no identifiable lesion for the active oozing that was discovered. INR was redrawn after multiple doses of vitamin K and fresh frozen plasma, which demonstrated minimal improvement. The patient began to have hematochezia, requiring multiple blood transfusions after endoscopy; so, a nuclear bleeding scan was performed and demonstrated active bleeding in the descending colon, as well as retained blood in the stomach and duodenum.

In the setting of numerous areas of gastrointestinal bleeds and coagulopathy refractory to multiple reversal agents, blood mixing studies were ordered. The partial thromboplastin time (PTT) and prothrombin time (PT) mixing studies revealed an inhibitor, which resulted in additional testing including factor 2, factor 5 and factor 10. Fibrinogen was normal which helped to exclude disseminated intravascular coagulation (DIC). Factors 2 and 10 were normal. Factor 5 was undetectable, which raised the suspicion for acquired factor V inhibition. Prednisone was started at the dose of 1 mg/kg to suppress factor V autoantibodies, but the patient continued to have coagulopathy and subsequent bleeding. The patient and his family eventually agreed to hospice, and he expired.

## 3. Discussion

Liver disease and specifically cirrhosis have unique hemostatic abnormalities, as the disease process is both pro-thrombotic and anticoagulant [6]. The liver plays a key role in both synthesis and post-translational modification of many proteins, so in a diseased state patients are at risk of bleeding and clotting due to abnormalities in protein structure and formation [7]. Thrombocytopenia from splenic sequestration and decreased hepatic synthesis of thrombopoietin leads to an increased likelihood of bleeding, just disruption in platelet adhesion and activation seen in the liver disease [3]. The liver also produces coagulation factors I, II, V, VII, IX, X, and XI just as with chronic liver disease or cirrhosis; it is expected to have decreased number and function of clotting factors, also making patients prone to bleeding [8]. Hyperfibrinolysis or the premature dissolution of fibrin clots is associated with liver disease and can lead to both bleeding and clotting [9].

Along with clotting factors, the liver also produces anticoagulants antithrombin III, protein c, and protein s; in chronic liver disease, these too will be decreased in a similar fashion. With decreased number and function of anticoagulants, the balance is shifted towards a pro-thrombotic dynamic [10]. Vascular stasis and endothelial inflammation and dysfunction are also common in liver disease and associated with increased thrombosis [11].

In the setting of cirrhosis, bleeding secondary to portal hypertensive complications like esophageal varices, portal hypertensive gastropathy, portal hypertensive duodenopathy and hemorrhoids is often seen and managed by hospitalists and gastroenterologists. In circumstances where patients have elevated Prothrombin time/INR, reversal agents like vitamin K, cryoprecipitate, fresh frozen plasma, or prothrombin complex concentrates are often administered to help control bleeding [12,13,14].

When these methods are used to no avail, additional work ups should be pursued to assess for acquired factor inhibitors. Factor inhibitors are antibodies that either cause destruction of coagulation factors or inhibit their functions [15]. Acquired factor V inhibition is extremely rare and typically related to previous exposure to fibrin glue or bovine thrombin during surgery. Autoimmune disease, certain malignancies, pregnancy, and the postpartum period are other causes of factor V inhibition that have been seen infrequently. Rarely, medications have been associated with factor V inhibition, specifically rifaximin and B-lactams like ceftriaxone, as was seen in this case where the patient was on rifaximin for prevention of portosystemic encephalopathy and ceftriaxone for spontaneous bacterial peritonitis prophylaxis [2,4].

Factor inhibitors should be suspected when practitioners are faced with refractory coagulopathy despite reversal therapies. Acquired factor V inhibition can clinically be suspected when patients have normal thrombin time, prolonged prothrombin time/INR, and prolonged activated partial thromboplastin time [16]. Mixing tests should then be performed and a diagnosis can be confirmed with low factor V levels [17]. Treatment of acquired factor V inhibition should include intravenous immunoglobulin and plasma exchange with or without platelet transfusion for severe bleeding [18]. Immunosuppression should also be considered for life-threatening bleeds including prednisone, cyclophosphamide and rituximab in refractory cases [19]. Although acquired factor V inhibition carries a great prognosis due to autoantibody clearance within a few months, patients must first survive the initial bleeding episode, which tends to carry high mortality rates [20].

The coagulopathy differential with both PTT and PT prolongation included liver disease, DIC, severe vitamin K deficiency, acquired inhibitors, anticoagulation and amyloidosis associated factor 10 deficiency. Vitamin K deficiency was ruled out with high and multiple dose vitamin K administration. Fibrinogen was normal, decreasing the suspicion for DIC and the patient was not receiving any anticoagulation. If the coagulopathy was related to liver disease, factors II, V, and X should all be decreased, but we only found an undetectable level in factor V. The patient did receive vitamin K along with FFP upon presentation to the emergency department, but the FFP did not skew the results of the undetectable factor 5. FFP has an indication for bleeding or expected bleeding in individuals with deficiencies of multiple coagulation factors such as liver disease, massive transfusion, anticoagulation with warfarin along with isolated factor 5 deficiencies. Patients receiving FFP would raise the factor 5 level in the absence of an inhibitor. FFP was administered 3 days prior to any factor levels being drawn.

The value of viscoelecastic testing has been reviewed by several trials in liver-related bleeding including a study with 96 individuals with transfusions guided by thromboelastrography (TEG) or by PT/INR and platelet count [21]. Both groups had similar control of bleeding, but the TEG group had fewer blood products and transfusion-related adverse events. There was no statistical significance on survival difference and the trial design has been questioned in the following literature. At this time, authors recommend further studies before directing care based on TEG. Other studies are ongoing, and it brings up an interesting consideration for future research and guidelines, but as of now, it is not standard of care to guide transfusions in cirrhotic patients with TEG. Currently, our hospital does not offer TEG testing or other testing similar to TEG.

This case report demonstrates the utility of mixing studies to identify factor inhibition in the setting of refractory coagulopathy, especially in cirrhotic patients whose coagulopathy is assumed to be related to liver disease alone. Identification of factor inhibitors early in the hospital course can lead to different treatment modalities, as was seen in this case when steroids were started. This case report also demonstrates how antibiotics, such as ceftriaxone and rifaximin are potential culprits for acquired factor inhibition. This is especially important as cirrhotic patients typically have coagulopathies and are also treated with these medications to prevent complications that are inherent to cirrhosis.

## References

[1] Bennett J., Cunningham M.T., Howard C., Hoffmann M., Plapp F.V. (2021). Acquired factor V inhibitor in the setting of coronavirus disease 2019 infection. Blood Coagul. Fibrinolysis.

[2] Franchini M., Lippi G. (2011). Acquired factor V inhibitors: A systematic review. J. Thromb. Thrombolysis.

[3] Bänninger H., Hardegger T., Tobler A., Barth A., Schüpbach P., Reinhart W., Lämmle B., Furlan M. (1993). Fibrin glue in surgery: Frequent development of inhibitors of bovine thrombin and human factor V. Br. J. Haematol..

[4] Hirai D., Yamashita Y., Masunaga N., Katsura T., Akao M., Okuno Y., Koyama H. (2016). Acquired Factor V Inhibitor. Intern. Med..

[5] Cui Q.Y., Shen H.S., Wu T.Q., Chen H.F., Yu Z.Q., Wang Z.Y. (2015). Development of acquired factor V inhibitor after treatment with ceftazidime: A case report and review of the literature. Drug Des. Devel. Ther..

[6] Tripodi A., Mannucci P.M. (2011). The coagulopathy of chronic liver disease. N. Engl. J. Med..

[7] Marks P.W. (2013). Hematologic manifestations of liver disease. Semin. Hematol..

[8] Northup P.G., Caldwell S.H. (2013). Coagulation in liver disease: A guide for the clinician. Clin. Gastroenterol. Hepatol..

[9] Wölpl A., Lattke H., Board P.G., Arnold R., Schmeiser T., Kubanek B., Robin-Winn M., Pichelmayr R., Goldmann S.F. (1987). Coagulation factor XIII A and B subunits in bone marrow and liver transplantation. Transplantation.

[10] Blasi A., Patel V.C., Adelmeijer J., Azarian S., Hernandez Tejero M., Calvo A., Fernández J., Bernal W., Lisman T. (2020). Mixed Fibrinolytic Phenotypes in Decompensated Cirrhosis and Acute-on-Chronic Liver Failure with Hypofibrinolysis in Those with Complications and Poor Survival. Hepatology.

[11] Sinegre T., Duron C., Lecompte T., Pereira B., Massoulier S., Lamblin G., Abergel A., Lebreton A. (2018). Increased factor VIII plays a significant role in plasma hypercoagulability phenotype of patients with cirrhosis. J. Thromb. Haemost..

[12] Palyu E., Harsfalvi J., Tornai T., Papp M., Udvardy M., Szekeres-Csiki K., Pataki L., Vanhoorelbeke K., Feys H.B., Deckmyn H. (2018). Major Changes of von Willebrand Factor Multimer Distribution in Cirrhotic Patients with Stable Disease or Acute Decompensation. Thromb. Haemost..

[13] Britt R.B., Brown J.N. (2018). Characterizing the Severe Reactions of Parenteral Vitamin K1. Clin. Appl. Thromb. Hemost..

[14] Pandit T.N., Sarode R. (2012). Blood component support in acquired coagulopathic conditions: Is there a method to the madness?. Am. J. Hematol..

[15] Tischendorf M., Fuchs A., Zeuzem S., Lange C.M. (2019). Use of prothrombin complex concentrates in patients with decompensated liver cirrhosis is associated with thromboembolic events. J. Hepatol..

[16] Franchini M., Gandini G., Di Paolantonio T., Mariani G. (2005). Acquired hemophilia A: A concise review. Am. J. Hematol..

[17] Lossing T.S., Kasper C.K., Feinstein D.I. (1977). Detection of factor VIII inhibitors with the partial thromboplastin time. Blood.

[18] Zehnder J.L., Leung L.L. (1990). Development of antibodies to thrombin and factor V with recurrent bleeding in a patient exposed to topical bovine thrombin. Blood.

[19] Knöbl P., Lechner K. (1998). Acquired factor V inhibitors. Baillieres Clin. Haematol..

[20] Navarrete M.A., van der Meer F.J., Damiani G., Diaz A., Eikenboom J. (2012). The use of rituximab therapy in patients with acquired factor V inhibitors. Am. J. Hematol..

[21] Kumar M., Ahmad J., Maiwall R., Choudhury A., Bajpai M., Mitra L.G., Saluja V., Mohan Agarwal P., Bihari C., Shasthry S.M. (2020). Thromboelastography-Guided Blood Component Use in Patients with Cirrhosis with Nonvariceal Bleeding: A Randomized Controlled Trial. Hepatology.

